# Supplementary material for: An artificial neural network classification method employing longitudinally monitored immune biomarkers to predict the clinical outcome of critically ill COVID-19 patients
Source: PeerJ. 2022 Dec 12;10:e14487. doi: 10.7717/peerj.14487 (PMC9753745; doi:10.7717/peerj.14487)
Supplement: Supplemental Information 1 — We provide a description of the individual function rationalizing upon the selection of each one of the 28 biomarkers considered in this study. We also provide the reference in which the function has been previously described. [file peerj-10-14487-s001.docx]

| **Biomarker** | **Function** | **Reference** |
| --- | --- | --- |
| ICAM1 | Its ligand role allows the binding stabilization between leukocytes and endothelial cells, facilitating leukocyte endothelial transmigration, serving as a mortality predictor for COVID-19 sepsis. | Pingatelli *et* al, 1994  Kaur *et* al, 2021 |
| LIPO | Sequestrates iron and prevent it to be used by bacteria for their growth. Also functions as a growth factor, stimulating immune cells proliferation. Its expression was found to be higher during sepsis onsets. | Yang *et* al, 2002  Vazquez *et* al, 2014 |
| MPO | Peroxidase is an enzyme that is expressed in neutrophil granulocytes. It produces hypohalous acid to be used in antimicrobial activities. Therefore, its upregulation is positively associated with cytokine storms and consequently, being a predictor of death. | Nichols and Hazen, 2005 |
| VCAM | Promotes the adhesion of immune cells to vascular endothelium. It was found to be elevated in COVID-19 patients (mild) and drastically elevated in severe manifestations. | Kong *et* al, 2018  Tong *et* al, 2020 |
| D-Dimer | It is a small product found in blood after the dissolution of blood clots. Elevated levels of it have been associated to predicting severity in COVID-19 infections. | Asakura and Ogawa, 2021  Lehmann *et* al, 2021 |
| E-selectin | Expressed by endothelial cells, this glycoprotein slows down leukocytes rolling, promoting the recruitment of immune cells to the endothelium. This protein was found to be a predictor of ICU admission in COVID-19 patients. | Timmerman *et* al, 2016  Oliva *et* al, 2021 |
| Ferritin | Increased ferritin levels represent a host immune mechanism that deprives iron-based bacterial growth. Critical COVID patients showed higher levels of it compared to mild, granting the predictive characteristic to the biomarker. | Kernan and Carcillo, 2017  Kaushal *et* al, 2022 |
| SPD | It is predominantly found in the lung surfactant. It promotes the aggregation of inhaled microorganisms, protecting the lung. Higher concentrations of it were suggested to be associated with severe lung injuries. | Sorensen, 2018  Tong *et* al, 2021 |
| PDL1 | It supresses the immune system by a binding interaction (with PD1), transmitting an inhibitory signal. It is upregulated in critical manifestations of COVID. | Ahmadzadeh *et* al, 2009  Sabbatino *et* al, 2021 |
| GCSF | Causes cells (mainly neutrophils) found in the bone marrow to become mature and activated, ready to circulate in the blood stream. | Brender *et* al, 2006 |
| IL1β | It is a cytokine that marks several cellular activities, such as cell proliferation, differentiation, and apoptosis. It also has a pro-inflammatory role. The suppression of IL1b in inflammatory states was potentially identified as having therapeutic effects in preventing respiratory failures caused by COVID-19. | Andenæs *et* al, 2018  Conti *et* al, 2020  Van de Veerdonk *et* al, 2020 |
| IL1ra | Inhibits the activities of IL1β. | Patterson *et* al, 2003 |
| VEGFC | Promotes the growth of new blood vessels, being involved in migration, growth, and differentiation of endothelial cells. Apart from angiogenesis, it also induces vascular leakiness and permeability, therefore, anti-VEGFC therapeutics are potential candidates for treatment in ICU patients. | Rauniyar *et* al, 2018  Sahebnasagh *et* al, 2021  Turkia 2020 |
| ANG2 | Promotes angiogenesis. In a context-dependent scenario, acts alongside with VEGFC to form new blood vessels. | Fangiani and Christofori, 2013 |
| CXCL10 | Chemoattractant for immune cells. Promotes the adhesion of T cells to endothelial cells. High levels of it were associated with predicting severity of COVID. | Lee *et* al, 2009  Lore *et* al, 2021 |
| GMCSF | It stimulates stem cells to produce granulocytes and monocytes. Increased concentrations of GMCSF were reported in COVID-19 patients compared to healthy controls. | Francisco-Cruz *et* al, 2014  Leavis *et* al, 2022 |
| IL10 | Downregulates the expression of cytokines, antigens, and stimulant factors, limiting the host immune response. It was found to drastically rise in severe cases of COVID-19. | Iyer and Cheng, 2012  Islam *et* al, 2021 |
| IL17a | Allows the maturation of non-mature immune cells. It also links activation of T cells to neutrophil mobilization. Interestingly, it was linked as a marker of disease progression as it activates inflammatory pathways, resulting in cytokine storms. | Zenobia and Hajishengallis, 2015  Maione *et* al, 2021 |
| IL6 | IL-6 maintain both pro- and anti-inflammatory cascade by binding with its soluble and cognate receptor respectively. Its upregulation marks the progression of severity in COVID-19 patients. | Scheller *et* al, 2011  Cruz *et* al, 2021 |
| IL7 | Stimulates cellular differentiation of hematopoietic stem cells into lymphoid progenitor cells. Therapies based on it were associated with restoration of lymphocytes, enhancing the antiviral activity. | Laterre *et* al, 2020  Chen *et* al, 2021 |
| CCL2 | Promotes the recruitment of monocytes, T cells, and dendritic cells to sites of inflammation. High levels of it correlates with activation of coagulation and respiratory impairment, related to pathology of COVID-19. | Carr *et* al, 1994  Nieri *et* al, 2021 |
| GRANB | It is found in the granules of immune cells. This enzyme is secreted to mediate apoptosis. GRANB inhibitors are potential targets for therapeutics, aiming to regulate the pro-inflammatory activity of the biomarker. | Velotti *et* al, 2020 |
| IFNɣ | Primary activator of macrophages and stimulant of natural killer cells and neutrophils. Its expression was found to be higher in immune cells of critically ill patients. | Schoenborn and Wilson, 2007  Gadotti *et* al, 2020 |
| IL12 | It activates T cells and natural killers; it also increases their toxicity. High levels of it were associated with severe progression of COVID-19. | Kaliński *et* al, 1997  Moll-Bernardes *et* al, 2021 |
| IL15 | It regulates the activation and proliferation of T cells and natural killers. It has a role in viral clearance and has been suggested as a potential immunotherapy. | Perera *et* al, 2012  Kandikattu *et* al, 2020 |
| IL2 | It is a T-cell growth factor. Its levels were found elevated in critical COVID-19 patients. | Jian *et* al, 2016  Shi *et* al, 2020 |
| IL4 | It induces the differentiation of naïve T cells into Th2 cells. It also regulates antibody production. When compared to H1N1 patients, higher levels of IL4 were found in COVID-19 induced lung injuries. | Gadani *et* al, 2013  Vaz de Paula *et* al, 2020 |
| TNF-ɑ | It mediates inflammatory responses by regulating growth and differentiation of several immune cells. Inhibitors of this protein were linked to a lower probability of hospitalization. | Idriss and Naismith, 2000.  Guo *et* al, 2022 |

References

Ahmadzadeh, M., Johnson, L. A., Heemskerk, B., Wunderlich, J. R., Dudley, M. E., White, D. E., & Rosenberg, S. A. (2009). Tumor antigen-specific CD8 T cells infiltrating the tumor express high levels of PD-1 and are functionally impaired. *Blood*, *114*(8). https://doi.org/10.1182/blood-2008-12-195792

Andenæs, R., Småstuen, M. C., Misvær, N., Ribu, L., Vistad, I., Helseth, S., Rahman, A., Jackson, H., Hristov, H., Isaacson, R. S., Saif, N., Shetty, T., Etingin, O., Henchcliffe, C., Brinton, R. D., Mosconi, L., Correa-Rodríguez, M., Mansouri-Yachou, J. el, Casas-Barragán, A., … Payam, F. (2018). Mayo Clinic - Pharmaceuticals &amp; Healthcare - Deals and Alliances Profile. *ProQuest Dissertations and Theses*, *17*(2).

Asakura, H., & Ogawa, H. (2021). COVID-19-associated coagulopathy and disseminated intravascular coagulation. *International Journal of Hematology*, *113*(1). https://doi.org/10.1007/s12185-020-03029-y

Brender E, Lynm C, Glass RM. Granulocyte-Colony Stimulating Factor. *JAMA.* 2006;295(9):1088. doi:10.1001/jama.295.9.1088

Carr, M. W., Roth, S. J., Luther, E., Rose, S. S., & Springer, T. A. (1994). Monocyte chemoattractant protein 1 acts as a T-lymphocyte chemoattractant. *Proceedings of the National Academy of Sciences of the United States of America*, *91*(9). https://doi.org/10.1073/pnas.91.9.3652

Chen, D., Tang, T. X., Deng, H., Yang, X. P., & Tang, Z. H. (2021). Interleukin-7 Biology and Its Effects on Immune Cells: Mediator of Generation, Differentiation, Survival, and Homeostasis. In *Frontiers in Immunology* (Vol. 12). https://doi.org/10.3389/fimmu.2021.747324

Conti, P., Ronconi, G., Caraffa, A., Gallenga, C. E., Ross, R., Frydas, I., & Kritas, S. K. (2020). Induction of pro-inflammatory cytokines (IL-1 and IL-6) and lung inflammation by Coronavirus-19 (COVI-19 or SARS-CoV-2): anti-inflammatory strategies. In *Journal of biological regulators and homeostatic agents* (Vol. 34, Issue 2). https://doi.org/10.23812/CONTI-E

Fagiani, E., & Christofori, G. (2013). Angiopoietins in angiogenesis. In *Cancer Letters* (Vol. 328, Issue 1). https://doi.org/10.1016/j.canlet.2012.08.018

Francisco-Cruz, A., Aguilar-Santelises, M., Ramos-Espinosa, O., Mata-Espinosa, D., Marquina-Castillo, B., Barrios-Payan, J., & Hernandez-Pando, R. (2014). Granulocyte-macrophage colony-stimulating factor: Not just another haematopoietic growth factor. *Medical Oncology*, *31*(1). https://doi.org/10.1007/s12032-013-0774-6

Gadani, Sachin P; Cronk, J. (2013). Interleukin-4: A Cytokine to Remember. *The Journal of Immunology*, *189*(9).

Gadotti, A. C., de Castro Deus, M., Telles, J. P., Wind, R., Goes, M., Garcia Charello Ossoski, R., de Padua, A. M., de Noronha, L., Moreno-Amaral, A., Baena, C. P., & Tuon, F. F. (2020). IFN-γ is an independent risk factor associated with mortality in patients with moderate and severe COVID-19 infection. *Virus Research*, *289*. https://doi.org/10.1016/j.virusres.2020.198171

Guo, Y., Hu, K., Li, Y., Lu, C., Ling, K., Cai, C., Wang, W., & Ye, D. (2022). Targeting TNF-α for COVID-19: Recent Advanced and Controversies. In *Frontiers in Public Health* (Vol. 10). https://doi.org/10.3389/fpubh.2022.833967

Idriss, H. T., & Naismith, J. H. (2000). TNFα and the TNF receptor superfamily: Structure-function relationship(s). *Microscopy Research and Technique*, *50*(3). [https://doi.org/10.1002/1097-0029(20000801)50:3<184::AID-JEMT2>3.0.CO;2-H](https://doi.org/10.1002/1097-0029(20000801)50:3%3c184::AID-JEMT2%3e3.0.CO;2-H)

Islam, H., Chamberlain, T. C., Mui, A. L., & Little, J. P. (2021). Elevated Interleukin-10 Levels in COVID-19: Potentiation of Pro-Inflammatory Responses or Impaired Anti-Inflammatory Action? *Frontiers in Immunology*, *12*. https://doi.org/10.3389/fimmu.2021.677008

Iyer, S. S., & Cheng, G. (2012). Role of interleukin 10 transcriptional regulation in inflammation and autoimmune disease. *Critical Reviews in Immunology*, *32*(1). https://doi.org/10.1615/critrevimmunol.v32.i1.30

Jiang, T., Zhou, C., & Ren, S. (2016). Role of IL-2 in cancer immunotherapy. In *OncoImmunology* (Vol. 5, Issue 6). <https://doi.org/10.1080/2162402X.2016.1163462>

Kandikattu, H. K., Venkateshaiah, S. U., Kumar, S., & Mishra, A. (2020). IL-15 immunotherapy is a viable strategy for COVID-19. In *Cytokine and Growth Factor Reviews* (Vol. 54). https://doi.org/10.1016/j.cytogfr.2020.06.008

Kaur, S., Hussain, S., Kolhe, K., Kumar, G., Tripathi, D. M., Tomar, A., Kale, P., Narayanan, A., Bihari, C., Bajpai, M., Maiwall, R., Gupta, E., & Sarin, S. K. (2021). Elevated plasma ICAM1 levels predict 28-day mortality in cirrhotic patients with COVID-19 or bacterial sepsis. *JHEP Reports*, *3*(4). https://doi.org/10.1016/j.jhepr.2021.100303

Kaushal, K., Kaur, H., Sarma, P., Bhattacharyya, A., Sharma, D. J., Prajapat, M., Pathak, M., Kothari, A., Kumar, S., Rana, S., Kaur, M., Prakash, A., Mirza, A. A., Panda, P. K., Vivekanandan, S., Omar, B. J., Medhi, B., & Naithani, M. (2022). Serum ferritin as a predictive biomarker in COVID-19. A systematic review, meta-analysis and meta-regression analysis. *Journal of Critical Care*, *67*. https://doi.org/10.1016/j.jcrc.2021.09.023

Kaliński, P., Hilkens, C. M., Snijders, A., Snijdewint, F. G., & Kapsenberg, M. L. (1997). IL-12-deficient dendritic cells, generated in the presence of prostaglandin E2, promote type 2 cytokine production in maturing human naive T helper cells. *Journal of Immunology (Baltimore, Md. : 1950)*, *159*(1).

Kernan, K. F., & Carcillo, J. A. (2017). Hyperferritinemia and inflammation. *International Immunology*, *29*(9). https://doi.org/10.1093/intimm/dxx031

Kong, D. H., Kim, Y. K., Kim, M. R., Jang, J. H., & Lee, S. (2018). Emerging roles of vascular cell adhesion molecule-1 (VCAM-1) in immunological disorders and cancer. In *International Journal of Molecular Sciences* (Vol. 19, Issue 4). https://doi.org/10.3390/ijms19041057

Laterre, P. F., François, B., Collienne, C., Hantson, P., Jeannet, R., Remy, K. E., & Hotchkiss, R. S. (2020). Association of Interleukin 7 Immunotherapy with Lymphocyte Counts among Patients with Severe Coronavirus Disease 2019 (COVID-19). *JAMA Network Open*, *3*(7). https://doi.org/10.1001/jamanetworkopen.2020.16485

Leavis, H. L., van de Veerdonk, F. L., & Murthy, S. (2022). Stimulating severe COVID-19: the potential role of GM-CSF antagonism. In *The Lancet Respiratory Medicine* (Vol. 10, Issue 3). https://doi.org/10.1016/S2213-2600(21)00539-7

Lee, E. Y., Lee, Z. H., & Song, Y. W. (2009). CXCL10 and autoimmune diseases. In *Autoimmunity Reviews* (Vol. 8, Issue 5). <https://doi.org/10.1016/j.autrev.2008.12.002>

Lehmann, A., Prosch, H., Zehetmayer, S., Gysan, M. R., Bernitzky, D., Vonbank, K., Idzko, M., & Gompelmann, D. (2021). Impact of persistent D-dimer elevation following recovery from COVID-19. *PLoS ONE*, *16*(10 October). https://doi.org/10.1371/journal.pone.0258351

Lorè, N. I., de Lorenzo, R., Rancoita, P. M. V., Cugnata, F., Agresti, A., Benedetti, F., Bianchi, M. E., Bonini, C., Capobianco, A., Conte, C., Corti, A., Furlan, R., Mantegani, P., Maugeri, N., Sciorati, C., Saliu, F., Silvestri, L., Tresoldi, C., Farina, N., … Manfredi, A. A. (2021). CXCL10 levels at hospital admission predict COVID-19 outcome: hierarchical assessment of 53 putative inflammatory biomarkers in an observational study. *Molecular Medicine*, *27*(1). https://doi.org/10.1186/s10020-021-00390-4

Maione, F., Casillo, G. M., Raucci, F., Salvatore, C., Ambrosini, G., Costa, L., Scarpa, R., Caso, F., & Bucci, M. (2021). Interleukin-17A (IL-17A): A silent amplifier of COVID-19. *Biomedicine and Pharmacotherapy*, *142*. https://doi.org/10.1016/j.biopha.2021.111980

Moll-Bernardes, R., de Sousa, A. S., Macedo, A. V. S., Lopes, R. D., Vera, N., Maia, L. C. R., Feldman, A., Arruda, G. D. A. S., Castro, M. J. C., Pimentel-Coelho, P. M., de Albuquerque, D. C., de Paula, T. C., Furquim, T. A. B., Loures, V. A., Giusti, K. G. D., de Oliveira, N. M., de Luca, F. A., Kotsugai, M. D. M., Domiciano, R. A. M., … Medei, E. (2021). IL-10 and IL-12 (P70) Levels Predict the Risk of Covid-19 Progression in Hypertensive Patients: Insights From the BRACE-CORONA Trial. *Frontiers in Cardiovascular Medicine*, *8*. https://doi.org/10.3389/fcvm.2021.702507

Nicholls, S. J., & Hazen, S. L. (2005). Myeloperoxidase and cardiovascular disease. In *Arteriosclerosis, Thrombosis, and Vascular Biology* (Vol. 25, Issue 6). https://doi.org/10.1161/01.ATV.0000163262.83456.6d

Nieri, D., Neri, T., Barbieri, G., Moneta, S., Morelli, G., Mingardi, D., Spinelli, S., Ghiadoni, L., Falcone, M., Tiseo, G., Menichetti, F., Franzini, M., Caponi, L., Paolicchi, A., Pancani, R., Pistelli, F., Carrozzi, L., & Celi, A. (2021). C-C motive chemokine ligand 2 and thromboinflammation in COVID-19-associated pneumonia: A retrospective study. *Thrombosis Research*, *204*. https://doi.org/10.1016/j.thromres.2021.06.003

Oliva, A., Rando, E., al Ismail, D., de Angelis, M., Cancelli, F., Miele, M. C., Aronica, R., Mauro, V., di Timoteo, F., Loffredo, L., & Mastroianni, C. M. (2021). Role of serum e-selectin as a biomarker of infection severity in coronavirus disease 2019. *Journal of Clinical Medicine*, *10*(17). https://doi.org/10.3390/jcm10174018

Patterson, D., Jones, C., Hart, I., Bleskan, J., Berger, R., Geyer, D., Eisenberg, S. P., Smith, M. F., & Arend, W. P. (1993). The human interleukin-1 receptor antagonist (IL1RN) gene is located in the chromosome 2q14 region. *Genomics*, *15*(1). https://doi.org/10.1006/geno.1993.1025

Perera, P. Y., Lichy, J. H., Waldmann, T. A., & Perera, L. P. (2012). The role of interleukin-15 in inflammation and immune responses to infection: Implications for its therapeutic use. In *Microbes and Infection* (Vol. 14, Issue 3). <https://doi.org/10.1016/j.micinf.2011.10.006>

Pignatelli, M., & Vessey BM, C. J. (1994). Adhesion molecules: Novel molecular tools in tumor pathology. *Human Pathology*, *25*(9). https://doi.org/10.1016/0046-8177(94)90002-7

Rauniyar, K., Jha, S. K., & Jeltsch, M. (2018). Biology of vascular endothelial growth factor C in the morphogenesis of lymphatic vessels. In *Frontiers in Bioengineering and Biotechnology* (Vol. 6, Issue FEB). https://doi.org/10.3389/fbioe.2018.00007

Sabbatino, F., Conti, V., Franci, G., Sellitto, C., Manzo, V., Pagliano, P., de Bellis, E., Masullo, A., Salzano, F. A., Caputo, A., Peluso, I., Zeppa, P., Scognamiglio, G., Greco, G., Zannella, C., Ciccarelli, M., Cicala, C., Vecchione, C., Filippelli, A., & Pepe, S. (2021). PD-L1 Dysregulation in COVID-19 Patients. *Frontiers in Immunology*, *12*. https://doi.org/10.3389/fimmu.2021.695242

Sahebnasagh, A., Nabavi, S. M., Kashani, H. R. K., Abdollahian, S., Habtemariam, S., & Rezabakhsh, A. (2021). Anti-VEGF agents: As appealing targets in the setting of COVID-19 treatment in critically ill patients. In *International Immunopharmacology* (Vol. 101). https://doi.org/10.1016/j.intimp.2021.108257

Santa Cruz, A., Mendes-Frias, A., Oliveira, A. I., Dias, L., Matos, A. R., Carvalho, A., Capela, C., Pedrosa, J., Castro, A. G., & Silvestre, R. (2021). Interleukin-6 Is a Biomarker for the Development of Fatal Severe Acute Respiratory Syndrome Coronavirus 2 Pneumonia. *Frontiers in Immunology*, *12*. https://doi.org/10.3389/fimmu.2021.613422

Scheller, J., Chalaris, A., Schmidt-Arras, D., & Rose-John, S. (2011). The pro- and anti-inflammatory properties of the cytokine interleukin-6. In *Biochimica et Biophysica Acta - Molecular Cell Research* (Vol. 1813, Issue 5). https://doi.org/10.1016/j.bbamcr.2011.01.034

Schoenborn, J. R., & Wilson, C. B. (2007). Regulation of Interferon-γ During Innate and Adaptive Immune Responses. In *Advances in Immunology* (Vol. 96). https://doi.org/10.1016/S0065-2776(07)96002-2

Shi, H., Wang, W., Yin, J., Ouyang, Y., Pang, L., Feng, Y., Qiao, L., Guo, X., Shi, H., Jin, R., & Chen, D. (2020). The inhibition of IL-2/IL-2R gives rise to CD8+ T cell and lymphocyte decrease through JAK1-STAT5 in critical patients with COVID-19 pneumonia. *Cell Death and Disease*, *11*(6). https://doi.org/10.1038/s41419-020-2636-4

Sorensen, G. L. (2018). Surfactant protein D in respiratory and non-respiratory diseases. In *Frontiers in Medicine* (Vol. 5, Issue FEB). https://doi.org/10.3389/fmed.2018.00018

Timmerman, I., Daniel, A. E., Kroon, J., & van Buul, J. D. (2016). Leukocytes Crossing the Endothelium: A Matter of Communication. In *International Review of Cell and Molecular Biology* (Vol. 322). <https://doi.org/10.1016/bs.ircmb.2015.10.005>

Tong, M., Xiong, Y., Zhu, C., Xu, H., Zheng, Q., Jiang, Y., Zou, L., Xiao, X., Chen, F., Yan, X., Hu, C., & Zhu, Y. (2021). Serum surfactant protein D in COVID-19 is elevated and correlated with disease severity. *BMC Infectious Diseases*, *21*(1). https://doi.org/10.1186/s12879-021-06447-3

Tong, M., Jiang, Y., Xia, D., Xiong, Y., Zheng, Q., Chen, F., Zou, L., Xiao, W., & Zhu, Y. (2020). Elevated expression of serum endothelial cell adhesion molecules in COVID-19 patients. *Journal of Infectious Diseases*, *222*(6). https://doi.org/10.1093/infdis/jiaa349

Turkia, M. (2020). COVID-19 As An Endothelial Disease and Its Relationship to Vascular Endothelial Growth Factor (VEGF) and Iodide. *SSRN Electronic Journal*. https://doi.org/10.2139/ssrn.3604987

van de Veerdonk, F. L., & Netea, M. G. (2020). Blocking IL-1 to prevent respiratory failure in COVID-19. In *Critical Care* (Vol. 24, Issue 1). https://doi.org/10.1186/s13054-020-03166-0

Vaz de Paula, C. B., de Azevedo, M. L. V., Nagashima, S., Martins, A. P. C., Malaquias, M. A. S., Miggiolaro, A. F. R. dos S., da Silva Motta Júnior, J., Avelino, G., do Carmo, L. A. P., Carstens, L. B., & de Noronha, L. (2020). IL-4/IL-13 remodeling pathway of COVID-19 lung injury. *Scientific Reports*, *10*(1). https://doi.org/10.1038/s41598-020-75659-5

Vazquez, D. E., Niño, D. F., de Maio, A., & Cauvi, D. M. (2015). Sustained expression of lipocalin-2 during polymicrobial sepsis. *Innate Immunity*, *21*(5). https://doi.org/10.1177/1753425914548491

Velotti, F., Barchetta, I., Cimini, F. A., & Cavallo, M. G. (2020). Granzyme B in Inflammatory Diseases: Apoptosis, Inflammation, Extracellular Matrix Remodeling, Epithelial-to-Mesenchymal Transition and Fibrosis. In *Frontiers in Immunology* (Vol. 11). <https://doi.org/10.3389/fimmu.2020.587581>

Yang, J., Goetz, D., Li, J. Y., Wang, W., Mori, K., Setlik, D., Du, T., Erdjument-Bromage, H., Tempst, P., Strong, R., & Barasch, J. (2002). An iron delivery pathway mediated by a lipocalin. *Molecular Cell*, *10*(5). https://doi.org/10.1016/S1097-2765(02)00710-4

Zenobia, C., & Hajishengallis, G. (2015). Basic biology and role of interleukin-17 in immunity and inflammation. *Periodontology 2000*, *69*(1). https://doi.org/10.1111/prd.12083
