# Supplementary material for: An artificial neural network classification method employing longitudinally monitored immune biomarkers to predict the clinical outcome of critically ill COVID-19 patients
Source: PeerJ. 2022 Dec 12;10:e14487. doi: 10.7717/peerj.14487 (PMC9753745; doi:10.7717/peerj.14487)
Supplement: Supplemental Information 4 — We assessed the accuracy of different algorithms to classify (i) the train/test dataset and (ii) the validation dataset. [file peerj-10-14487-s004.docx]

| **Model** | **Train/test accuracy (%)** | **Validation accuracy (%)** |
| --- | --- | --- |
| SVM | 84 | 54 |
| KNN | 65 | 57 |
| CART | 77.7 | 54 |
| RF | 91 | 60 |
| XGBoost | 91 | 47 |
| Deep learn ANN | 93 | 85.5 |
