# Supplementary material for: An artificial neural network classification method employing longitudinally monitored immune biomarkers to predict the clinical outcome of critically ill COVID-19 patients
Source: PeerJ. 2022 Dec 12;10:e14487. doi: 10.7717/peerj.14487 (PMC9753745; doi:10.7717/peerj.14487)
Supplement: Supplemental Information 5 — We assessed the error drop rate in different artificial neural network architectures. Up to four hidden layers were considered with 5, 10, 15, and 20 neurons each. [file peerj-10-14487-s005.docx]

| Hidden layers | Neurons per hidden layer | Error (train/test) | Validation (recovered) | Validation (deceased) |
| --- | --- | --- | --- | --- |
| 1 | 5 | 0.4500 | 0.50 | 0.25 |
| 2 | 5 | 0.2529 | 0.60 | 0.40 |
| 3 | 5 | 0.4272 | 0.66 | 0.45 |
| 4 | 5 | 0.3180 | 0.60 | 0.55 |
| 1 | 10 | 0.1666 | 0.45 | 0.60 |
| 2 | 10 | 0.1000 | 0.70 | 0.50 |
| 3 | 10 | 0.0715 | 0.74 | 0.45 |
| 4 | 10 | 0.0133 | 0.63 | 0.60 |
| 1 | 15 | 0.1104 | 0.66 | 0.60 |
| 2 | 15 | 0.0924 | 0.60 | 0.55 |
| 3 | 15 | 0.0096 | 0.70 | 0.63 |
| 4 | 15 | 0.0056 | 0.60 | 0.60 |
| 1 | 20 | 0.1096 | 0.63 | 0.50 |
| 2 | 20 | 0.0332 | 0.66 | 0.50 |
| 3 | 20 | 0.0042 | 0.70 | 0.76 |
| 4 | 20 | 0.0020 | 0.83 | 0.88 |
